# Effect of Oral + Topical and Only Topical Tranaxamic Acid Application on Blood Loss and Postoperative Transfusion in Primary Total Hip Arthroplasty

**DOI:** 10.3390/jcm14041275

**Published:** 2025-02-14

**Authors:** Tansel Mutlu, Mehmet Arıcan, Zekeriya Okan Karaduman, Yalçın Turhan, İlyas Kaban, Raşit Emin Dalaslan, Sönmez Sağlam, Mücahid Osman Yücel

**Affiliations:** 1 Department of Orthopedics and Traumatology, Gebze Medical Park Hospital, Kocaeli 41400, Türkiye; tanselmutlu@yahoo.com; 2 Department of Orthopedics and Traumatology, Faculty of Medicine, Duzce University, Duzce 81620, Türkiye; ari_can_mehmet@hotmail.com (M.A.); karadumano@hotmail.com (Z.O.K.); dr.sonmezsaglam@gmail.com (S.S.); mucahidosmanyucel@gmail.com (M.O.Y.); 3 Department of Orthopedics and Traumatology, Bilkent City Hospital, Health Sciences University, Ankara 06800, Türkiye; yturhan_2000@yahoo.com; 4 Department of Orthopedics and Traumatology, Duzce Ataturk State Hospital, Duzce 81010, Türkiye; drilyaskaban@gmail.com

**Keywords:** tranexamic acid, arthroplasty, replacement, hip

## Abstract

**Objectives**: Total hip arthroplasty is one of the most common procedures performed to reduce pain and improve hip functions in patients with advanced hip osteoarthritis, but perioperative blood loss, acute anemia and transfusion requirement increase the risk of morbidity and mortality during and after surgery and negatively affect functional recovery. We aimed to present the comparative results of oral + topical and only topical tranexamic acid application to reduce blood loss and postoperative transfusion in primary total hip arthroplasty. **Methods**: We retrospectively evaluated the patients who applied to the Orthopedics and Traumatology outpatient clinic with complaints of hip pain and limited movement between January 2014 and December 2020, who underwent primary total hip arthroplasty with the diagnosis of coxarthrosis and who were administered oral + topical and only topical tranexamic acid before and during surgery, in terms of blood loss and transfusion requirement. **Results**: No statistically significant difference was observed between the preoperative, day 0 and day 1 hemoglobin means in those that were applied oral + topical tranexamic acid and those that were applied only topical (*p* > 0.05). However, the second- and third-day hemoglobin means in those that were treated with topical medication alone were found to be statistically significantly lower than in those that were treated with oral + topical tranexamic acid (*p* = 0.032, *p* = 0.0001). **Conclusions**: Oral + topical tranexamic acid application in total hip arthroplasty surgery is more effective than topical applications alone when it comes to reducing blood loss, hemoglobin and hematocrit decrease without increasing the risk of thromboembolic diseases and wound complications.

## 1. Introduction

Total hip arthroplasty (THA) is an orthopedic procedure that is frequently used to treat advanced hip osteoarthritis [1]. However, perioperative blood loss during THA treatment can cause complications such as acute anemia, need for transfusion, prolonged hospitalization, and increased mortality [2]. Studies have shown that the amount of blood loss in patients undergoing primary THA treatment ranges from 700 mL to 2000 mL, and 16% to 37% of these patients require transfusion [3].

There are many different methods of reducing blood loss during the perioperative period. Some of these include good bleeding control during surgery, application of bone wax to the cut surface after femoral neck cutting, use of hemostatic agents such as oxidized regenerated cellulose (SURGICEL^®^) and carbazochrome sodium sulfonate (CSS), and use of antifibrinolytic agents such as aminocaproic acid and tranexamic acid [4,5,6,7]. One of the most prominent of these is tranexamic acid application. Tranexamic acid is used to reduce blood loss in various surgical procedures by inhibiting fibrinolysis and activating plasminogen [8]. Tranexamic acid can be administered intravenously, topically, and orally, but there is no clear consensus on the superiority of these applications over each other [1,9,10,11,12,13].

Studies comparing oral and intravenous administration have reported similar results in terms of perioperative blood loss [1,12]. In some studies, it has been observed that bleeding values change positively with the addition of topical application in addition to intravenous application [3,8]. There are studies showing that topical tranexamic acid application alone is effective in reducing bleeding [9,14]. When intravenous application alone is compared with topical application alone, it has been shown that intravenous application is more effective than topical application in terms of reducing blood loss [14].

In this study, we hypothesized whether oral + topical and topical tranexamic acid alone are superior to each other in reducing blood loss in patients undergoing primary total hip arthroplasty, and we aimed to contribute to the literature by publishing the comparative results.

## 2. Materials and Methods

This clinical study was conducted with the approval of Düzce University Non-Interventional Health Research Ethics Committee numbered 2020/194; the approval date was 17 August 2020.

This study is a retrospective study that included the data of patients with primary coxarthrosis who applied to the Orthopedics and Traumatology clinic with complaints of hip pain and limited movement between January 2014 and January 2020 and who underwent primary total hip arthroplasty. The study included 160 patients aged between 20 and 80 years who underwent cementless total hip arthroplasty due to unilateral primary coxarthrosis, who were administered perioperative oral + topical tranexamic acid or only intraoperative topical tranexamic acid and who gave their consent to participate in the study. Patients who underwent additional surgery, had a history of arterial embolism, had renal problems, had cardiovascular problems, or were allergic to tranexamic acid were excluded from the study.

The patients were divided into two groups: group 1 (n = 81) administered oral + topical tranexamic acid and group 2 (n = 79) administered only topical tranexamic acid. Routine oral tranexamic acid application in primary total hip arthroplasty cases started in our clinic in 2018. Cases which began before this date were in the group that received only topical application, and cases which began after this date were in the group that received both oral and topical application.

All total hip arthroplasty procedures were performed by the same surgical team using the same surgical technique. A standard lateral decubitus position and posterolateral approach were used in all cases. Cementless acetabular cups and cementless femoral stems were used in all cases. Topical tranexamic acid was applied to both groups included in the study during surgery. For topical application, a solution was created by adding 1 g of tranexamic acid (Transamine, Actavis, Istanbul, Turkey) to 100 mL of normal saline. This solution was applied intraoperatively in three stages. The first application was to prepare the acetabulum and then wash the acetabulum surface with 20 mL of solution. In the second stage, the femoral canal was reamed and then washed with 20 mL of solution. In the third stage, the remaining 60 mL of solution was applied into the joint after the fascia was closed during the closure stage, and the drain was kept closed for 30 min. This topical application method was found to be safe in previous studies [8,13,14]. In addition to topical application, oral tranexamic acid (Transamine, Actavis, Istanbul, Turkey) was also applied to the patients in group 1. As an oral administration procedure, 4 tablets containing a total of 2 g of tranexamic acid were administered at once, 2 h before the start of surgery. Pharmacokinetic studies have shown that 2 g of oral tranexamic acid administered at once reaches therapeutic levels approximately 2 h later and maintains levels above the therapeutic threshold for 6 h after administration [12,15].

As a standard procedure in our clinic, all patients received intravenous prophylactic antibiotics for 24 h. Antiembolic stockings were worn on both lower extremities after surgery for deep vein thrombosis (DVT) prophylaxis. They were mobilized from the first postoperative day. As a chemoprophylaxis procedure, 0.4 mL of low-molecular-weight heparin (LMWH; 4000 IU in 0.4 mL; Clexane, Sanofi-Aventis, France) was administered once daily for 20 days, starting at the 8th hour postoperatively [16,17]. Blood transfusion criteria were determined as hemoglobin levels <70 g/L or 70–100 g/L with symptomatic anemia (dizziness, fatigue that prevents participation in treatment, palpitations, or shortness of breath not related to another cause) [17,18].

Patients’ height, weight, gender, side, age, American Society of Anesthesia (ASA) scores, anesthesia method applied during surgery, amount of postoperative drainage, amount of postoperative transfusion, complications, whether DVT developed, comorbidities, operation time, hemoglobin and hematocrit values on preoperative and postoperative days 0, 1, 2 and 3 were recorded. Drains were removed on the first postoperative day. Doppler ultrasound was performed for symptomatic patients for DVT evaluation. Patients who underwent total hip arthroplasty were routinely followed up for 3 months.

Our primary outcomes in this study are the decrease in hemoglobin and hematocrit values and the amount of drainage in the postoperative period, and our secondary outcomes are the postoperative transfusion rate and DVT rate.

The statistical analyses in this study were performed with the NCSS (Number Cruncher Statistical System) 2007 Statistical Software version 1 (Salt Lake City, UT, USA) package program. In the evaluation of the data, in addition to descriptive statistical methods (mean, standard deviation), the distribution of variables was examined with the Shapiro–Wilk normality test, paired one-way analysis of variance was used in time comparisons of normally distributed variables, Newman–Keuls’ multiple comparison test was used in subgroup comparisons, an independent t-test was used in comparisons between paired groups, and a chi-square test was used in comparisons of qualitative data. The results were evaluated at the significance level of *p* < 0.05. Considering a similar study, the sample size was calculated based on a Type I error (0.05) and targeted power (0.80), and it was concluded that the total sample size should be at least 158 people (G*Power3.1.9.4) [1].

## 3. Results

The results of 160 patients who underwent primary total hip arthroplasty in our clinic between January 2014 and January 2020 were evaluated. They were divided into two groups: 81 patients who received both oral and topical tranexamic acid (group 1) and 79 patients who received only topical tranexamic acid (group 2). The patients’ age, gender, body mass index (BMI), side, ASA scores, anesthesia types, operation time, drainage amount, whether or not a transfusion was performed, presence of comorbidities, whether or not complications developed, and whether or not DVT developed are shown in Table 1, which is organized by group.

There was no statistically significant difference between the two groups in terms of mean age, gender, body mass index, side, ASA score, type of anesthesia applied, and operation time (Table 1). In group 2, where only topical tranexamic acid was applied, the amount of drainage was found to be significantly higher than in group 1 (Table 1). Similarly, the rate of transfusion was significantly higher in group 2 than in group 1 (Table 1). In group 1, nine patients received 1 unit of blood transfusion on postoperative day 0, and four patients received 1 unit of blood transfusion on postoperative day 1. In group 2, 16 patients received 1 unit of blood transfusion on postoperative day 0, and 9 patients received 1 unit of blood transfusion on postoperative day 1, and 1 patient in group 2 received a total of 2 units of blood transfusion on both days 0 and 1.

No significant difference was observed between the two groups in terms of comorbidity and complication rates (Table 1). In terms of the comorbidities, hypertension was identified in 13 patients, rheumatic diseases were reported in 5 patients, diabetes was reported in 5 patients, infectious diseases (hbv, hcv) were reported in 2 patients, hypothyroidism was reported in 1 patient, and 1 patient had a previous history of breast cancer. Since they were not directly related to bleeding, additional statistical calculations were not performed. Similarly, complications included leg length difference, prosthesis dislocation, superficial skin infections, and peripostatic fractures. Since these were not directly related to bleeding and tranexamic acid treatment, no additional statistical calculations were performed. No significant difference was observed between the two groups in terms of the rate of patients developing DVT (Table 1).

**Figure 1:**
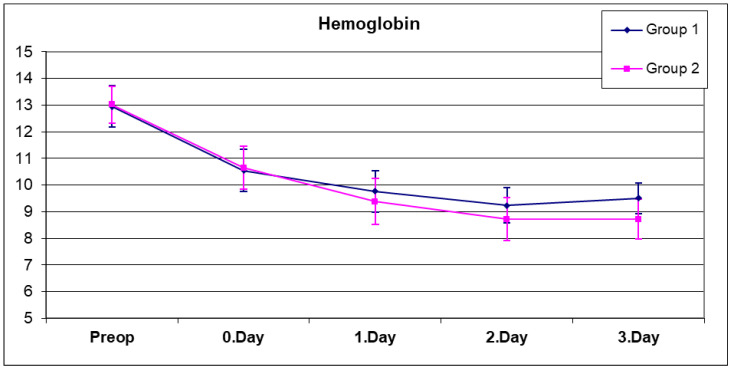
Graph containing average hemoglobin values of both groups.

**Figure 2:**
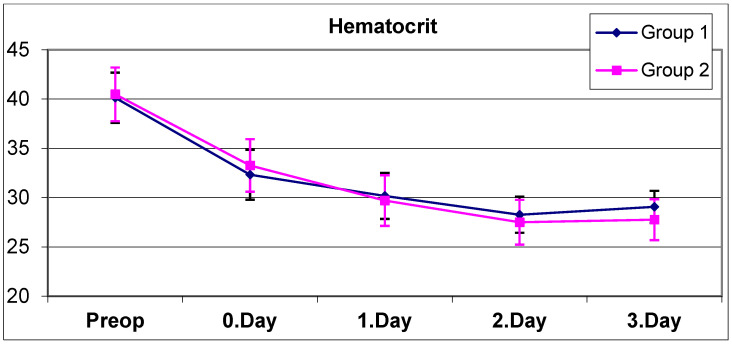
Graph containing the average hematocrit values of both groups.

A complete blood count was performed five times for each patient, preoperatively, on the same day after surgery (day 0), and on postoperative days 1, 2, and 3 (Figure 1 and Figure 2). The mean hemoglobin values of both groups are as shown in Table 2. No significant difference was observed between the preoperative, day 0, and day 1 hemoglobin values of both groups (Table 2) (*p* > 0.05). When the hemoglobin values on the second day were compared, the mean hemoglobin value in group 2, which was composed of patients who received only topical tranexamic acid, was found to be significantly lower than in group 1 (Table 2) (*p* = 0.032). Similarly, when the hemoglobin values on the third day were compared, the mean hemoglobin value of group 2 was found to be significantly lower than in group 1 (Table 2) (*p* = 0.0001).

A statistically significant change was observed between the preoperative, day 0, day 1, day 2, and day 3 hemoglobin means of group 1 (Table 2) (*p* = 0.0001). As expected, due to the effect of blood loss during surgery and in the early period, a significant decrease in hemoglobin values was observed on postoperative day 0, day 1, and day 2, but this decrease stopped on day 3, and in fact, the hemoglobin value on day 3 exceeded the value on day 2 in group 1. This may be related to the fact that excessive fluid replacement therapy applied in the first days of postoperative period was generally stopped on the second and third days.

A statistically significant change was observed between the preoperative, day 0, day 1, day 2 and day 3 hemoglobin means of group 2 (Table 2) (*p* = 0.0001). In group 2, the hemoglobin decrease continued on day 0, day 1, and day 2, but this decrease stopped on day 3. No statistically significant difference was found between the postoperative hemoglobin values on day 2 and 3 (*p* = 0.999).

The mean hematocrit values of all patients preoperatively and on postoperative day 0, day 1, day 2 and day 3 are shown in Table 3. No statistically significant difference was observed between the preoperative, day 0, day 1, and day 2 hematocrit means of group 1 and group 2 (Table 3) (*p* > 0.05). The mean hematocrit of group 2 on day 3 was found to be statistically significantly lower than the mean hematocrit of group 1 on day 3 (Table 3) (*p* = 0.028).

A statistically significant change was observed between the preoperative, day 0, day 1, day 2, and day 3 hematocrit means of group 1 (Table 3) (*p* = 0.0001). The significant decrease in hematocrit values of group 1 continued on day 0, day 1, day 2, and day 3 in the controls. Although the decrease in hemoglobin stopped on day 3, probably due to IV fluid replacement, the decrease in the hematocrit value continued on day 3.

A statistically significant change was observed between the preoperative, day 0, day 1, day 2, and day 3 hematocrit means of group 2 (Table 3) (*p* = 0.0001). In group 2, a significant decrease in hematocrit values continued to occur on postoperative days 0, 1, and 2, but this decrease stopped on day 3. No statistically significant difference was observed between the hematocrit averages on day 2 and day 3 (*p* = 0.236).

The changes in mean hemoglobin and hematocrit values compared to the preoperative values in both groups are shown in Table 4.

No statistically significant difference was observed between the preoperative day 0 mean hemoglobin changes in group 1 and group 2 (Table 4) (*p* = 0.765). The preoperative day 1 mean hemoglobin change in group 2 was found to be statistically significantly higher than the preoperative day 1 mean hemoglobin change in group 1 (Table 4) (*p* = 0.012). The preoperative day 2 mean hemoglobin change in group 2 was found to be statistically significantly higher than the preoperative day 2 mean hemoglobin change in group 1 (Table 4) (*p* = 0.003). The preoperative day 3 mean hemoglobin change in group 2 was found to be statistically significantly higher than the preoperative day 3 mean hemoglobin change in group 1 (Table 4) (*p* = 0.0001).

No statistically significant difference was observed between the changes in preoperative/day 0, preoperative/day 1, and preoperative/day 2 mean hematocrit values of group 1 and group 2 (Table 4) (*p* > 0.05). The change in the preoperative/day 3 mean hematocrit values of group 2 was found to be statistically significantly higher than the change in the preoperative/day 3 mean hematocrit values of group 1 (Table 4) (*p* = 0.017).

## 4. Discussion

Tranexamic acid treatment is a method that is frequently used to reduce bleeding-related complications during total hip arthroplasty surgery. Studies have proven that tranexamic acid provides a significant reduction in bleeding due to its antifibrinolytic properties [1,3,8]. However, although there are many relevant studies in the literature, there is still no consensus on the application method.

In a randomized clinical study conducted by Cao G. et al., 108 patients who underwent primary total hip arthroplasty were evaluated [1]. Intravenous tranexamic acid was administered to each patient group in the preoperative period at 20 mg/kg, but oral tranexamic acid was administered to one group at 3 × 2 g and intravenous tranexamic acid was administered to the other group at 3 × 1 g in the postoperative period. In the results, total blood loss, occult blood loss and hemoglobin decreases on the first and second days were evaluated and the results were found to be similar in both groups, and no statistically significant difference was observed between them [1]. According to this study, the clinical results of continuing oral or intravenous tranexamic acid administration in the postoperative period after a single dose of preoperative intravenous tranexamic acid were similar.

In a meta-analysis study published by Liu X. et al. in 2017, 747 patients who underwent primary total hip arthroplasty were evaluated [3]. In this study, the results of intravenous tranexamic acid application were compared with both intravenous and topical tranexamic acid application. The results showed that the addition of topical tranexamic acid application to intravenous tranexamic acid application during surgery resulted in a 250 mL decrease in total blood loss, an 117 mL decrease in occult blood loss, and a 9.1% decrease in transfusion rate. However, no statistically significant difference was observed between hemoglobin decreases, hospital stays, and DVT development rates [3]. In the results of this study, the combined application of topical tranexamic acid in addition to intravenous application provided a significant decrease in blood loss and a decrease in transfusion rates. Similarly, in our study, it was observed that the hemoglobin values on the second and third postoperative days in patients who received combined application of oral tranexamic acid and topical tranexamic acid were significantly higher than in the group that received only topical tranexamic acid.

Similarly, in a randomized controlled study conducted by Yi Z. et al., the effects of intravenous tranexamic acid administration were compared with intravenous and topical 1 g tranexamic acid administration in a population of 150 patients [8]. In this study, it was observed that topical application added to intravenous administration reduced total blood loss, but there was no significant difference in the rate of DVT development [8].

A scan of the literature did not reveal any studies comparing topical application with oral + topical application, as was the case in our study; the closest examples we can look at are studies comparing systemic application (intravenous) with combined topical application with topical application only. In the prospective randomized double-blind study conducted by Palija S. et al. on this subject, 200 patients were divided into five groups and compared. Group 1 was the control group that did not receive tranexamic acid, group 2 comprised patients who were administered 2 g of intravenous tranexamic acid only, group 3 comprised patients who were administered 2 g of topical tranexamic acid only, group 4 comprised patients who were administered 2 g of combined tranexamic acid (1 g intravenous + 1 g topical) and group 5 was the group that was administered 4 g of combined (2 g intravenous + 2 g topical) application. According to the results of this study, it was observed there was less total blood loss in combined applications compared to topical applications only, which was similar to our study findings. When the group that received a total of 4 g of combined tranexamic acid (2 g intravenous + 2 g topical) was compared with the group that received only 2 g of topical tranexamic acid, total blood loss was found to be statistically significantly lower in the combined application group (*p* = 0.000) [19].

In a randomized clinical study conducted by Yue C. et al., the effects of high-dose (3 g) topical tranexamic acid application were evaluated in 101 patients who underwent primary total hip arthroplasty [9]. The results showed that topical tranexamic acid application alone reduced transfusion rates from 22.4% to 5.7% (*p* < 0.05), whereas there was no significant difference in the rates of complications such as pulmonary embolism and DVT. In addition, it was observed that total blood loss, blood loss with drainage, hemoglobin, and hematocrit decreases were statistically significantly lower in the topical tranexamic acid group [9].

In a retrospective study by Irwin A. et al., which involved 3000 cases, the results of oral tranexamic acid administration were compared with intravenous tranexamic acid administration in patients undergoing total hip and total knee arthroplasty [11]. A single dose of 15 mg/kg was administered intravenously at the beginning of anesthesia administration, and a single dose of 25 mg/kg was administered orally. The results showed that blood transfusion rates were significantly lower in the oral tranexamic acid group (*p* = 0.019). There was no significant difference in complication rates and hospital stays. According to the results of this study, oral tranexamic acid administration also provides effective results when compared to intravenous tranexamic acid administration, and it can be said that oral administration is a more cost-effective solution, as an additional benefit.

Similarly, in a randomized controlled study conducted by Kayupov E. et al. on 83 patients, the effects of oral (1.95 g tablet) tranexamic acid and intravenous (1 g) tranexamic acid administration were evaluated among patients undergoing total hip arthroplasty [12]. In the results of this study, it was determined that there was no significant difference between postoperative hemoglobin decreases, total blood losses, and transfusion rates.

In a retrospective study conducted by Wind TC. et al., which included 1494 patients who underwent total hip arthroplasty, patients were examined in three groups: those who were administered intravenous tranexamic acid, those who were administered only topical tranexamic acid, and those who were not administered tranexamic acid at all (control) [14]. The transfusion rates were evaluated and were found to be 19.86% for the control group and 4.39% for patients who underwent intravenous administration; this was significantly lower than in the control group (*p* < 0.001). In patients who were administered only topical tranexamic acid, the transfusion rate was found to be 12.86% in the control group, although its statistical significance was low (*p* = 0.15). According to the results of this study, it can be said that isolated topical tranexamic acid applications are not as effective as systemic tranexamic acid applications.

Tranexamic acid is frequently and routinely used via intravenous, oral, or topical application, but in a study conducted by Grassin-Delyle et al., intramuscular application was also evaluated [20]. In a study conducted on 15 volunteers, oral 2 g tablets, intravenous 1 g application, and intramuscular 1 g application were compared. The median peak concentration in the blood after intravenous application was measured as 57.5 mg/L, while the median peak concentration in the blood after intramuscular application was measured as 34.4 mg/L, and the median peak concentration after oral application was measured as 12.8 mg/L. In addition, when the times taken to reach the target tranexamic acid concentration of 10 mg/L in the blood were compared, a time of 3.5 min was found in intramuscular application and a time of 66 min was found in oral application. When these results are evaluated, it can be said that intramuscular tranexamic acid application gives very similar results to intravenous application in terms of the peak concentration in the blood and the time taken to reach the target concentration and is an alternative to intravenous application with its ease of application.

There are many different studies in the literature relating to the methods of tranexamic acid application. Different studies have shown that different methods are superior to each other or have similar results. It does not seem possible to reach a clear consensus on this issue with the existing studies. However, when the literature is examined in general, it can be said that oral application is as effective as intravenous application in reducing blood loss and related transfusions. However, it can be said that topical application alone is not as effective as intravenous and oral application. According to the results of our study and similar studies in the literature, it is thought that the combined application of oral and topical tranexamic acid would be more beneficial than the application of topical tranexamic acid alone, as postoperative hemoglobin decreases are smaller in patients undergoing primary total hip arthroplasty and it reduces transfusion rates.

Our study has some limitations. The study is retrospective, so randomization (which would have been ideal) could not be performed. Since the amount of bleeding during surgery was not recorded, the exact amount of bleeding is unknown, which is another limitation of our study. Postoperative drainage amounts were recorded, but this drainage may not have been entirely composed of blood; serous body fluids may have mixed into the drainage at different rates for each patient, which is another limitation of the study. This can be analyzed in future studies. The fact that this was a single-center study is also a limitation that makes it difficult to generalize the results. Conducting multicenter prospective studies on this subject will be useful in reaching accurate results in the future.

## 5. Conclusions

In conclusion, according to the data obtained from this study, oral + topical tranexamic acid application in total hip arthroplasty surgery was found to be more effective than topical applications alone with regard to reducing blood loss, hemoglobin and hematocrit decrease without increasing the risk of thromboembolic diseases and wound complications. However, multicenter prospective studies are required to reach more definitive conclusions.

## Figures and Tables

**Table 1:** Demographic and clinical data of both groups.

		Group 1		Group 2		*p*
Age		54.96 ± 12.29		54.84 ± 10.07		0.943 *
Gender	Male	34	41.98%	33	41.77%	0.979+
	Female	47	58.02%	46	58.23%	
BMI		25.77 ± 5.74		26.77 ± 4.87		0.241 *
Side	Right	50	61.73%	50	63.29%	0.838+
	Left	31	38.27%	29	36.71%	
ASA	1	12	14.81%	4	5.06%	0.162+
	2	40	49.38%	40	50.63%	
	3	26	32.10%	29	36.71%	
	4	3	3.70%	6	7.59%	
Anesthesia	General Anesthesia	10	12.35%	18	22.78%	0.082+
	Spinal Anesthesia	71	87.65%	61	77.22%	
Operation time		69.01 ± 9.82		69.37 ± 8.45		0.807 *
Drainage		217.28 ± 75.07		305.7 ± 119.56		0.0001 *
Transfusion	Not applied	68	83.95%	51	64.56%	0.005+
	Applied	13	16.05%	28	35.44%	
Comorbid	No	63	77.78%	70	88.61%	0.067+
	Yes	18	22.22%	9	11.39%	
Complication	No	72	88.89%	68	86.08%	0.591+
	Yes	9	11.11%	11	13.92%	
DVT	No	80	98.77%	77	97.47%	0.545+
	Yes	1	1.23%	2	2.53%	

* Independent *t*-test; + Chi Square test.

**Table 2:** Mean hemoglobin values of both groups.

Hemoglobin	Group 1	Group 2	*p* *
Preoperative	12.95 ± 1.54	13.02 ± 1.39	0.785
0.Day	10.55 ± 1.58	10.65 ± 1.60	0.677
1.Day	9.77 ± 1.55	9.38 ± 1.72	0.126
2.Day	9.23 ± 1.33	8.72 ± 1.63	0.032
3.Day	9.50 ± 1.13	8.72 ± 1.50	0.0001
*p* ^‡^	0.0001	0.0001	

* Independent *t*-test; ^‡^ paired one-way ANOVA.

**Table 3:** Mean hematocrit values of both groups.

Hematocrit	Group 1	Group 2	*p* *
Pre	40.13 ± 5.10	40.48 ± 5.45	0.681
0.Day	32.32 ± 5.07	33.26 ± 5.33	0.256
1.Day	30.17 ± 4.66	29.70 ± 5.14	0.540
2.Day	28.26 ± 3.66	27.50 ± 4.55	0.248
3.Day	29.06 ± 3.26	27.76 ± 4.15	0.028
*p* ^‡^	0.0001	0.0001	

* Independent *t*-test; ^‡^ paired one-way ANOVA.

**Table 4:** Changes in the mean hemoglobin and hematocrit values in both groups.

		Group 1	Group 2	*p* *
Hemoglobin	Pre-0.Day	2.40 ± 0.85	2.36 ± 0.91	0.765
	Pre-1.Day	3.18 ± 1.02	3.64 ± 1.26	0.012
	Pre-2.Day	3.72 ± 1.06	4.29 ± 1.35	0.003
	Pre-3.Day	3.45 ± 1.10	4.29 ± 1.30	0.0001
Hematocrit	Pre-0.Day	7.82 ± 2.99	7.22 ± 3.61	0.259
	Pre-1.Day	9.96 ± 3.30	10.78 ± 4.32	0.178
	Pre-2.Day	11.88 ± 3.69	12.98 ± 4.70	0.101
	Pre-3.Day	11.07 ± 3.86	12.72 ± 4.73	0.017

* Independent *t*-test.

## Data Availability

The original contributions presented in this study are included in the article. Further inquiries can be directed to the corresponding author.

## Abbreviations

The following abbreviations are used in this manuscript:

THA	Total hip arthroplasty.
DVT	Deep vein thrombosis.
LMWH	Low-molecular-weight heparin.
ASA	American Society of Anesthesia
NCSS	Number Cruncher Statistical System
BMI	Body mass index

## References

[1] Cao G., Huang Z., Xie J., Huang Q., Xu B., Zhang S., Pei F. (2018). The effect of oral versus intravenous tranexamic acid in reducing blood loss after primary total hip arthroplasty: A randomized clinical trial. Thromb. Res..

[2] Loftus T.J., Spratling L., Stone B.A., Xiao L., Jacofsky D.J. (2016). A Patient Blood Management Program in Prosthetic Joint Arthroplasty Decreases Blood Use and Improves Outcomes. J. Arthroplast..

[3] Liu X., Liu J., Sun G. (2017). A comparison of combined intravenous and topical administration of tranexamic acid with intravenous tranexamic acid alone for blood loss reduction after total hip arthroplasty: A meta-analysis. Int. J. Surg..

[4] Wakasa J., Iwakiri K., Ohta Y., Minoda Y., Kobayashi A., Nakamura H. (2024). Perioperative bleeding control in total hip arthroplasty: Hemostatic powder vs. tranexamic acid-a prospective randomized controlled trial. Arch. Orthop. Trauma Surg..

[5] Ye S., Chen M., Luo Y., Zhao C., Li Q., Kang P. (2023). Comparative study of carbazochrome sodium sulfonate and tranexamic acid in reducing blood loss and inflammatory response following direct anterior total hip arthroplasty: A prospective randomized controlled trial. Int. Orthop..

[6] Zheng C., Ma J., Xu J., Wu L., Wu Y., Liu Y., Shen B. (2022). The optimal regimen, efficacy and safety of tranexamic acid and aminocaproic acid to reduce bleeding for patients after total hip arthroplasty: A systematic review and Bayesian network meta-analysis. Thromb. Res..

[7] Mortazavi S.M.J., Razzaghof M., Ghadimi E., Seyedtabaei S.M.M., Ardakani M.V., Moharrami A. (2022). The Efficacy of Bone Wax in Reduction of Perioperative Blood Loss in Total Hip Arthroplasty via Direct Anterior Approach: A Prospective Randomized Clinical Trial. J. Bone Jt. Surg..

[8] Yi Z., Bin S., Jing Y., Zongke Z., Pengde K., Fuxing P. (2016). Tranexamic Acid Administration in Primary Total Hip Arthroplasty: A Randomized Controlled Trial of Intravenous Combined with Topical Versus Single-Dose Intravenous Administration. J. Bone Jt. Surg..

[9] Yue C., Kang P., Yang P., Xie J., Pei F. (2014). Topical Application of Tranexamic Acid in Primary Total Hip Arthroplasty: A Randomized Double-Blind Controlled Trial. J. Arthroplast..

[10] Imai N., Dohmae Y., Suda K., Miyasaka D., Ito T., Endo N. (2012). Tranexamic Acid for Reduction of Blood Loss During Total Hip Arthroplasty. J. Arthroplast..

[11] Irwin A., Khan S.K., Jameson S.S., Tate R.C., Copeland C., Reed M.R. (2013). Oral versus intravenous tranexamic acid in enhanced-recovery primary total hip and knee replacement: Results of 3000 procedures. Bone Jt. J..

[12] Kayupov E., Fillingham Y.A., Okroj K., Plummer D.R., Moric M., Gerlinger T.L., Della Valle C.J. (2017). Oral and Intravenous Tranexamic Acid Are Equivalent at Reducing Blood Loss Following Total Hip Arthroplasty. J. Bone Jt. Surg..

[13] Zeng Y., Si H., Shen B., Yang J., Zhou Z., Kang P., Pei F. (2017). Intravenous Combined with Topical Administration of Tranexamic Acid in Primary Total Hip Arthroplasty: A Randomized Controlled Trial. Orthop. Surg..

[14] Wind T.C., Barfield W.R., Moskal J.T. (2014). The Effect of Tranexamic Acid on Transfusion Rate in Primary Total Hip Arthroplasty. J. Arthroplast..

[15] Pilbrant Å., Schannong M., Vessman J. (1981). Pharmacokinetics and bioavailability of tranexamic acid. Eur. J. Clin. Pharmacol..

[16] Zaghiyan K.N., Sax H.C., Miraflor E., Cossman D., Wagner W., Mirocha J., Gewertz B., Fleshner P., Cedars-Sinai DVT Study Group (2016). Timing of Chemical Thromboprophylaxis and Deep Vein Thrombosis in Major Colorectal Surgery: A Ran-domized Clinical Trial. Ann. Surg..

[17] Xie J., Ma J., Yao H., Yue C., Pei F. (2016). Multiple Boluses of Intravenous Tranexamic Acid to Reduce Hidden Blood Loss After Primary Total Knee Arthroplasty Without Tourniquet: A Randomized Clinical Trial. J. Arthroplast..

[18] Zha G.-C., Zhu X.-R., Wang L., Li H.-W. (2022). Tranexamic acid reduces blood loss in primary total hip arthroplasty performed using the direct anterior approach: A one-center retrospective observational study. J. Orthop. Traumatol..

[19] Palija S., Bijeljac S., Manojlovic S., Jovicic Z., Jovanovic M., Cvijic P., Dragicevic-Cvjetkovic D. (2021). Effectiveness of different doses and routes of administration of tranexamic acid for total hip replacement. Int. Orthop..

[20] Grassin-Delyle S., Semeraro M., Lamy E., Urien S., Runge I., Foissac F., Bouazza N., Treluyer J.-M., Arribas M., Roberts I. (2022). Pharmacokinetics of tranexamic acid after intravenous, intramuscular, and oral routes: A prospective, randomised, crossover trial in healthy volunteers. Br. J. Anaesth..

